# Differential Expression of *HER2* and *SKP2* in Benign and Malignant Colorectal Lesions 

**DOI:** 10.31557/APJCP.2020.21.8.2357

**Published:** 2020-08

**Authors:** Mona Moussa, Afkar Badawy, Noha Helal, Fatma Hegab, Magdy Youssef, Tarek Aboushousha, Lubna Al Farouk, Dalal Elwy

**Affiliations:** 1 *Department of Pathology, Theodor Bilharz Research Institute, Imbaba, Giza, Egypt. *; 2 *Department of Gastroenterology and Hepatology, Theodor Bilharz Research Institute, Imbaba, Giza, Egypt. *; 3 *Department of Pathology, Faculty of Medicine, Cairo University, Giza, Egypt. *

**Keywords:** Immunohistochemistry, insitu hybridization, HER2, SKP2, benign, malignant, colonic lesions

## Abstract

**Background:**

Colorectal cancer (CRC) is the fourth most common cancer worldwide. Both HER2 and SKP2 have a carcinogenic role in CRC making them attractive targets for tailored treatment. This work aims to correlate HER2 and SKP2 protein expression as well as* HER2* gene amplification with clinicopathological parameters aiming at identifying potential candidates for targeted therapy.

**Methods:**

This Study was conducted on 127 paraffin-embedded tissue samples of different colorectal lesions [controls, chronic colitis, ulcerative colitis (UC), hyperplastic polyps (HPs), adenomas and CRCs] to investigate HER2 and SKP2 expression by immunohistochemistry (IHC), Selected CRC cases [equivocal (2+) and positive (3+) by IHC] were further evaluated by ISH (CISH and SISH ) to assess *HER2* gene amplification.

**Results:**

Chronic colitis, UC, HPs and adenomas were HER2-negative. HER2 positivity (scores 2+ and 3+) was found only in15% of CRCs. Both SISH and CISH showed the same results with high concordance as 66.7% of equivocal and 100% of positive cases showed amplification of* HER2* gene. SKP2 positivity was detected in 26.7% and 45% of adenomas and CRCs respectively, while other studied groups were negative. A significant correlation was noted between HER2 and SKP2 expression.

**Conclusion:**

A small percent of CRCs exhibited *HER2* gene amplification, which would be potential candidates for anti HER2 therapy whereas IHC could be a primary screening test for patient selection. A potential carcinogenic role of SKP2 was suggested by the findings that SKP2 expression was undetectable in normal colonic mucosa but significantly increases from adenoma to carcinoma, hoping adenoma patients to get benefit from targeted therapy.

## Introduction

Worldwide, colorectal cancer (CRC) ranks fourth in terms of incidence and second in terms of mortality (Bray et al., 2018). In Egypt, CRC constitutes 2.7% of diagnosed cancer patient and ranks the eighth most common cancer in both sexes and ninth cause of cancer-related deaths (Globoscan, 2018). The median age is 50 years; with younger cases usually complicate polyposis and ulcerative colitis (El-Bolkainy et al., 2013). The outcome of CRC patients has significantly improved over the past decades, reflecting continuous progress in understanding its biology, epidemiology, prevention, early diagnosis and treatment (Siegel et al., 2017), but the identification of clinically actionable oncogenic drivers and related predicted biomarkers are largely elusive (Zhang et al., 2020). 

CRC is a tumor that develops from the progression of acquired or hereditary premalignant lesions. It arises from interactions among different risk factors (environmental, dietary, familial and hereditary) that become relevant during the different stages of colorectal carcinogenesis (Jemal et al., 2011). Various genetic alterations and distinct molecular phenotypes for each tumor influence patient’s prognosis and response to chemotherapy. The choice of treatment depends on the identification of these particular molecular phenotypes. Targeted therapy is more efficient in responders and avoids unnecessary side-effects in non-responders (Hagan, Orr and Doyle 2013).

HER2 is a well-recognized mediator of the carcinogenic process. It has a role in a wide range of solid tumors, mainly via protein overexpression and/or gene amplification, thus making HER2 an attractive target for tailored treatment (Sartore-Bianchi et al., 2016; Wakatsuki et al., 2018). Although there have been many reports on the frequency of HER2 over-expression in colon cancer, the role of this oncogene in such cancer is not clearly defined (Erik et al., 2013). The most commonly used method to determine HER2 status is immunohistochemistry (IHC), which is a low-cost technique that can be performed on small samples, even formalin-fixed and paraffin-embedded tissues. Fluorescent in situ hybridization (FISH) is considered the gold standard and can be used to analyze this type of sample. An alternative is provided by the use of other in situ hybridization methods such as silver in situ hybridization (SISH) and chromogen in situ hybridization (CISH) which allows the use of an ordinary light microscope and has shown excellent correlation with results obtained using FISH (Abrahão-Machado et al., 2013). As the accurate assessment of *HER2* gene amplification status in CRC appears to be particularly important for patients who might undertake this specific targeted therapy (Zhang et al., 2020).

S-phase kinase-associated protein 2 (SKP2) belongs to the F-box protein family. SKP2 has been shown to regulate cellular proliferation by targeting several cell cycle-regulated proteins for degradation, including cyclin-dependant kinase inhibitor p27. SKP2 has also been demonstrated to display an oncogenic function since it’s over expression has been observed in many human cancers including CRC (Shapira et al., 2005). SKP2 may be a promising therapeutic target for colorectal cancer, and development of SKP2 inhibitors would have a great impact on colorectal cancer therapy (Bochis et al., 2015).

This work aims to correlate HER2 and SKP2 protein expression as well as *HER2* gene amplification with clinicopathological parameters aiming at identifying potential candidates for targeted therapy.

## Materials and Methods

*Specimens*

The material of this study were collected from 127 histologically documented cases with different colonic lesions from archives of Surgical Pathology Departments of Theodor Bilharz Research Institute (TBRI) and Faculty of Medicine, Cairo University, Egypt in the period from January 2015 to October 2017. The specimens were obtained either as endoscopic biopsies (109) or resection (colectomy) specimens (18). Specimens consisted of 40 CRCs, 15 adenomas, 15 ulcerative colitis, 19 chronic non-specific colitis, 8 hyperplastic polyps, 8 bilharzial colitis and 22 controls. 

*Histopathological Examination*

Serial sections were cut from paraffin blocks and stained with hematoxylin and eosin for routine histological examination. CRC classification, grading and staging were carried out following the 2017 AJCC staging system (Jessup et al., 2017). According to TNM classification (Edge et al., 2010), in colectomy specimens (n:18), CRC were classified as 12 specimens in T3 and 6 in T4; 6 in N0, 6 in N1 and 6 cases in N2. 

*Immunohistochemical (IHC) Technique*

Four-µm thick sections from formalin-fixed, paraffin-embedded colonic tissue were cut on charged slides. Antigen retrieval was performed with 10 ml sodium citrate buffer, pH 6.0, at 90°C for 30 min. Sections were incubated in 0.03% hydrogen peroxide for 10 min at room temperature, then in blocking serum (0.04% bovine serum albumin, A2153, Sigma-Aldrich, Shanghai, China), and 0.5% normal goat serum X0907, Dako Corporation, Carpinteria, CA, USA, in PBS) for 30 min at room temperature. Polyclonal antibodies for HER2/neu (A0485 Dako Denmark) and SKP2 (Chongqing Biospes Co., Ltd China) were applied at an optimal dilution of 1:700 and 1:200 respectively and incubated overnight at 4°C. Staining was developed with diaminobenzidine substrate and sections were counterstained with hematoxylin.

For each setting, positive and negative control slides were included. As a negative control, colon biopsy was processed in the above mentioned sequences but PBS was added instead of the primary antibody. 

Breast duct carcinoma and high grade prostate carcinoma were used as internal positive controls for HER2 and SKP2 respectively.

*Interpretation of immunostaining*

Expression of HER2 was assessed according to three criteria: (1) the pattern of staining, (2) intensity of staining and (3) percentage of stained colon cells. Pattern: membranous, cytoplasmic/membranous or cytoplasmic. Intensity: weak, moderate or strong. Percentage of stained cells: ≤10% stained cells= score 0, 10-40 % stained cells = score 1+. Both 0 and 1+ scores were considered negative, 40-70% stained cells = score 2+, which was considered equivocal, and >70% stained cells = score 3+, which was considered positive (Shabbir et al., 2016).

SKP2 was expressed as nuclear or cytoplasmic staining. Cases with more than 20% positively stained colon cells were considered SKP2 positive (Ni et al., 2009). 

*Insitu Hybridization (ISH) technique*

Equivocal and positive HER2 cases were evaluated for *HER2* gene status by insitu hybridization which were silver in situ hybridization (SISH) and chromogen in situ hybridization (CISH) for detection and confirmation of *HER2* gene amplification especially in cytoplasmic expressed cases. In each setting, a case negative for IHC HER2 staining (score 0/1+) was enrolled as a control.

*Silver Insitu Hybridization (SISH) technique and interpretation*

The BenchMark Series Automated Slide Stainer with HER2 Dual ISH DNA Probe Cocktail, reagents from UltraView Red ISH DIG and UltraView SISH DNP Detection Kits (Roche Tissue Diagnostics, VENTANA Medical Systems, USA) were used.* HER2* gene was presented by a black signal, while chromosome 17 was presented by a red signal. Tumor cells were scanned for hot spots by using x20 or x40 objectives and the area with the highest signal was selected. The signals were counted in 20 non-overlapping tumor cell nuclei from each case using x40 or x100 objectives. Small or large clusters were considered to be 6 signals and 12 signals respectively.

*HER2* gene amplification was defined as detection of *HER2* gene /CEP 17 ratio of ≥2 in 20 tumor nuclei. Normal colon epithelial cells and other adjacent benign cells served as internal controls (Valtorta et al., 2015).

*Chromogen insitu hybridization (CISH) technique and interpretation*

Manual HER2 CISH staining was done in Tissue Culture Lab, National Cancer Institute, Cairo, Egypt using Vortex the ZytoDot 2C SPEC ERBB2/CEN 17 Probe Kit (ZytoVision; Germany). A minimum number of 20 tumor cells per sample were evaluated. A red signal presents chromosome 17 and a green signal presents *HER2* gene. Tumor cells were scanned for hot spots by using x20 or x40 objectives and the area with the highest signal was selected. The signals were counted in 20 non-overlapping tumor cell nuclei from each case using x40 or x100 objectives. Small or large clusters were considered to be 6 signals and 12 signals respectively. 

*HER2* gene amplification was defined as detection of *HER2* gene /CEP 17 ratio of ≥2 in 20 tumor nuclei. Normal colon epithelial cells and other adjacent benign cells served as internal controls (Heppner et al., 2014 and Hanna et al., 2014). 

*Statistical Analysis*

SPSS software version 23 was used for statistical analysis (IBM Corporation, Armonk, New York, USA). Quantitative data were presented as mean + SD. For comparison of more than three groups; One way ANOVA test was used. Comparison between percent positive cases and staining intensity were calculated by Chi-Square test. Correlations between variables were studied using Spearmann’s correlation test. P value < 0.05 was considered statistically significant.

## Results

The study was performed on 105 specimens of colorectal lesions and 22 controls. Patient and groups characteristics are listed in Table 1.

*HER2 protein overexpression by IHC analysis*

**Figure 1:**
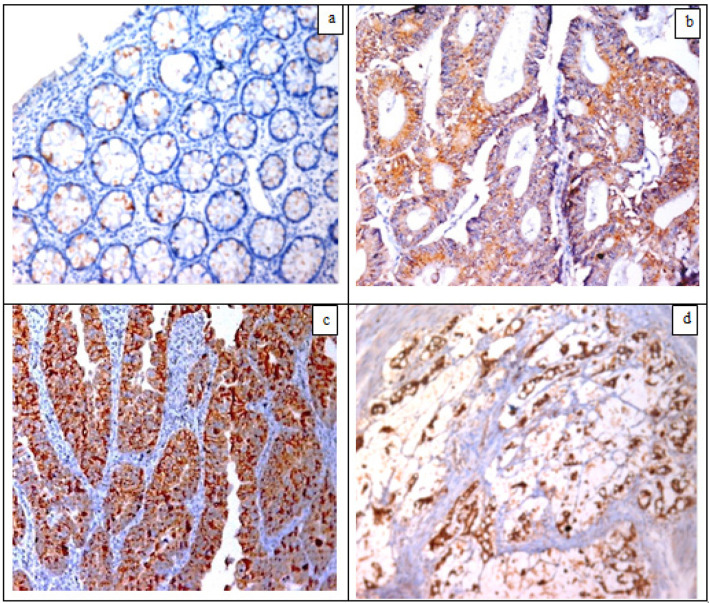
Immunohistochemistry for HER2 in Colonic Sections: (a) A case of mild colitis served as a control, negative for HER2 (IHCX200), (b) Low grade CRC, conventional type, G1,T3,N0, moderate cytoplasmic HER2 expression (IHCX200), (c) CRC on top of tubulovillous adenoma, conventional type, G2,T4,N0, strong membranous and cytoplasmic HER2 expression (IHC X200), (d) CRC, mucinous type, T3,N2, cytoplasmic HER2 expression (IHC X200)

HER2 immunoreactivity was detected as cytoplasmic, membranous or both expressions. Among these forty CRC tumors, HER2 IHC scores of 3+ (positive), 2+ (equivocal) and 0/1+ (negative) were observed in 3 (7.5%), 3(7.5%) and 34 (85%) tumors respectively. CRC cases expressing equivocal and positivity for HER2 showed statistically significant difference compared to the other groups (p<0.05) which were all negative for HER2 expression (Figure 1) (Table 2). 

In both conventional and mucinous subtypes of CRC, 6.3% and 9.3% of conventional subtype were of 3+ and 2+ score respectively compared to 12.5% of mucinous cases with 3+ score, without statistical significant difference (p=0.580). In addition, 8.3% of high grade conventional type showed 3+ score compared to 5% and 15% of low grade ones showed 3+ and 2+ score respectively without statistical significant difference. 

Regarding tumor depth and nodal metastasis, HER2 positivity, pattern of expression and intensity of expression do not show statistical significant difference in relation to different T and N stages (Table 3). 

Equivocal (2+ score) and positive (3+ score) CRC specimens showed cytoplasmic expression in 2/6 cases (33.3%) while the remaining 4/6 cases (66.7%) showed cytoplasmic/membranous expression, however, no significant relation between pattern of HER2 expression and each of subtypes, grade and stage of CRCs (Tables 2 and 3).

Out of 6 positive CRC cases, 4 showed strong intensity of HER2 immunoreactivity, all of conventional subtype with no statistical significant difference between studied grades or stages of tumor (Tables 2 and 3).

*Correlation between HER2 Protein Overexpression and HER2 Gene Amplification*

**Figure 2:**
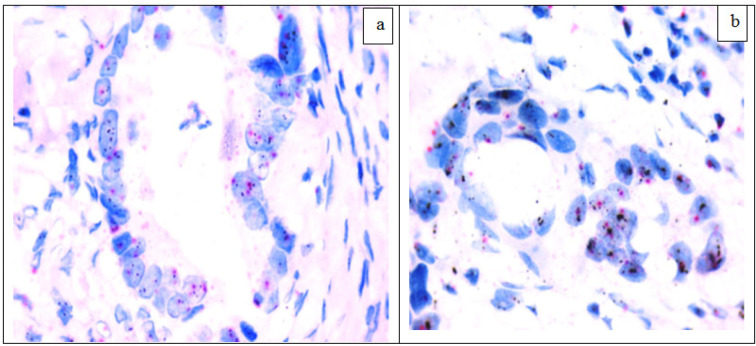
H*ER2* Silver Insitu Hybridization (a) CRC, Amplified HER2 gene; ratio between black dots (HER2 gene)and red dots (CEN 17) ≥2 (SISH x1000), (b) CRC, Amplified HER2 gene; aggregates of black dots (SISH x1000)

**Figure 3:**
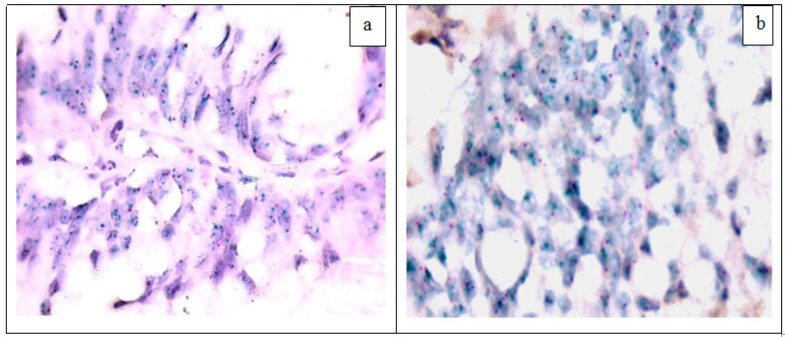
HER2 Chromogen Insitu Hybridization: (a) CRC, Amplified HER2 gene; ratio between green dots (HER2 gene) and red dots (CEN 17) ≥2 (CISH x1000), (b) CRC, Amplified HER2 gene; presence of small clusters of green dots (CISH x1000)

Specimens with 2+ (equivocal) and 3+ (positive) HER2 by IHC staining totally 6) were further evaluated by Silver Insitu Hybridization (SISH) and Chromogen Insitu Hybridization (CISH) to assay for *HER2* gene amplification. Both techniques showed the same results as 2/3 (66.7%) of equivocal and 3/3 (100%) of positive cases showed amplification of* HER2* gene with HER2/CEP 17 ratio ≥2 or clusters of* HER2* gene in >20% of cells. The control cases showed non amplified *HER2* gene (Figures 2 and 3) (Table 4).

*SKP2 Immunoexpression*

**Figure 4:**
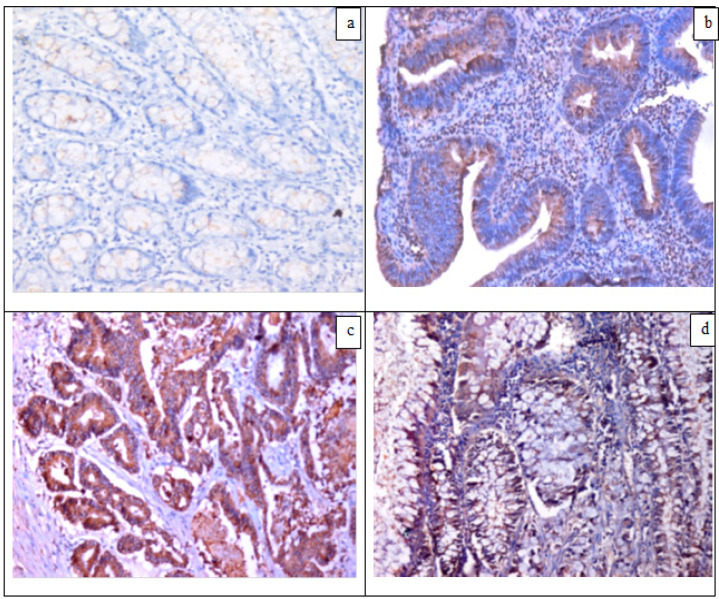
Immunohistochemistry for SKP2 in Colonic Sections: (a) A case of mild colitis served as a control, negative for SKP2 (IHCX200), (b) Tubular adenoma, cytoplasmic SKP2 expression (IHCX200), (c) CRC, conventional type,G2, cytoplasmic SKP2 expression (IHCX200), (d) CRC, mucinous type, cytoplasmic and nuclear SKP2 expression (IHC X400)

SKP2 immunoreactivity was found in 18/40 (45%) of CRCs and 4/15 (26.7%) of adenomas with high statistical significant difference compared to the other groups (p=0.000) which were negative for SKP2 (Figure 4). While 15/32 (46.9%) of conventional CRC cases were positive for SKP2; 3/8 (37.5%) of mucinous subtype were positive (p=0.634). In conventional subtype, 8/12 (66.7%) of high grade tumors were positive for SKP2 compared to 7/20 (35%) of low grade tumors without statistical significance. Also expression of SKP2 in different tumor stages was statistically insignificant (Table 5 and 6).

Regarding expression of SKP2 in adenoma, no statistical significant difference was detected between both types (tubular and tubulovillous) (p=0.310) nor between grades of associated dysplasia (Table 5).

SKP2 staining pattern was cytoplasmic, nuclear or both. In SKP2 positive CRC cases, 6/18 (33.3%) showed cytoplasmic expression, while 12/18 (66.7%) showed cytoplasmic/nuclear expression without statistical significant difference (P=0.746). 9/15 (60%) and 3/3 (100%) of conventional and mucinous subtypes respectively showed cytoplasmic/nuclear expression without statistical significant difference (Table 5). Cytoplasmic/nuclear expression was the predominant pattern in T3, N0, N1 and N2 (85.7%, 60%, 100% and 66.7% respectively) (Table 6). Positive adenomas showed cytoplasmic expression in 1/4 (25%) and cytoplasmic/ nuclear in 3/4 (75%) of positive cases (Table 5). 

Evaluated by spearman correlation test, we found significant positive correlation between HER2 and SKP2 immunopositivity among studied cases (r= 0.388, p=0.000).

**Table 1:** Clinico-Pathological Features of Studied Cases

Histopathological diagnosis (N)	Subtypes (N)	Gender	Age (years)
		(M/F) (%)	Mean ± SD
			(age range)
Adenocarcinoma (CRC) (40)	Conventional low	M: 19 (47.5%)	55.0 ± 12.77
	grade (20)	F: 21 (52.5%)	(28 - 77)
	Conventional high		
	grade (12)		
	Mucinous		
	adenocarcinoma (8)		
Adenoma (15)	With Low grade	M: 7 (46.7%)	58.58 ± 6.74
Tubular type (7)	dysplasia (6)	F: 8 (53.3%)	(52 - 75)
Tubulo-villous (8)	With high grade		
	dysplasia (9)		
Ulcerative colitis (UC) (15)	With low grade	M: 9 (60%)	34.08± 11.62^a,c^
	dysplasia (7)	F: 6 (40%)	(18 - 60)
	No dysplasia (8)		
Colorectal hyperplastic polyps (8)		M: 6 (75%)	52.50± 13.38
		F: 2 (25%)	(24 - 68)
Chronic non- specific	Moderate colitis (13)	M: 11 (57.9%)	37.94± 20.19^a,c ^
colitis (19)	Severe colitis (6)	F: 8 (42.1%)	(15 - 65)
Bilharzial colitis (8)		M: 6 (75%)	47.14 ± 14.18^b ^
		F: 2 (25%)	(31 – 67)
Total number of cases (105)		M: 58 (55.2%)	
		F: 47 (44.8%)	
Minimal colitis / controls (22)		M: 15(68.2%)	44.91 ± 15.71^a,b^
		F: 7 (31.8%)	(19 - 75)

N, number of cases; %. Percentage of cases; M, Male; F, Female; SD, standard deviation; CRC, colorectal adenocarcinoma; UC, ulcerative colitis; ^a^p<0.01 compared to adenoma and adenocarcinoma; ^b^p<0.05 compared to UC; ^c^p<0.05 compared to polyps

**Table 2:** HER2 Immunoreactivity Scoring among Studied Groups

Histopathological diagnosis (N)	Subtypes (N)		HER2 expression			HER2 pattern		HER2 intensity	
			Negative (0/1+) N (%)	Equivocal (2+) N (%)	Positive (3+) N (%)	Cytoplasmic N (%)	Cytoplasmic/membranous N (%)	Moderate N (%)	Strong N (%)
Adenocarcinoma (CRC) (40)			34 (85%)	3 (7.5%)a	3 (7.5%)^a^	2 (33.3%)	4 (66.7%)	2 (33.3%)	4 (66.7%)
	Conventional		27 (84.4%)	3 (9.4%)	2 (6.3%)	1 (20%)	4 (80%)	1 (20%)	4 (80%)
		Low grade (20)	16 (80%)	3 (15%)	1 (5%)	1 (25%)	3 (75%)	1 (25%	3 (75%)
		High grade (12)	11 (91.7%)	0	1 (8.3%)	0	1 (100%)	0	1 (100%)
	Mucinous (8)		7 (87.5%)	0	1 (12.5%)	1 (100%)	0	1 (100%)	0
Adenoma (15)	With Low grade		15 (100%)	0	0	-	-	-	-
Tubular type (7)	dysplasia (6)								
Tubulo-villous (8)	With high grade								
	dysplasia (9)								
Ulcerative colitis (UC) (15)	With low grade		15 (100%)	0	0	-	-	-	-
	dysplasia (7)								
	No dysplasia (8)								
Colorectal hyperplastic polyps (8)			8 (100%)	0	0	-	-	-	-
Chronic non- specific	Moderate colitis (13)		19 (100%)	0	0	-	-	-	-
colitis (19)	Severe colitis (6)								
Bilharzial colitis (8)			8 (100%)	0	0	-	-	-	-
Minimal colitis / controls (22)			22 (100%)	0	0	-	-	-	-

N, number of cases; %. Percentage of cases; CRC, colorectal adenocarcinoma; ^a^Crosstabs, Pearson chi square (p<0.05) between adenocarcinoma and other groups

**Table 3:** HER2 Immunoreactivity in Resection CRC Cases

Stage		HER2 expression				HER2 pattern			HER2 intensity		
		Negative (0/1+) N (%)	Equivocal (2+) N (%)	Positive (3+) N (%)	*P *	Cyto-plasmic N (%)	Cytoplas-mic/mem-branous N (%)	*P *	Moderate N (%)	Strong N (%)	*P *
T	T3 (12)	10 (83.30%)	1 (8.30%)	1 (8.30%)	0.725	1 (50%)	1 (50%)	0.248	1 (50%)	1 (50%)	1
	T4 (6)	4 (66.70%)	1 (16.70%)	1 (16.7%)		0	2 (100%)		1 (50%)	1 (50%)	
N	N0 (6)	4 (66.70%)	1 (16.70%)	1 (16.7%)	0.71	0	2 (100%)	0.135	1 (50%)	1 (50%)	0.368
	N1 (6)	5 (83.30%)	1 (16.70%)	0		0	1 (100%)		0	1 (100%)	
	N2 (6)	5 (83.3%)	0 (0%)	1 (16.7%)		1 (100%)	0		1 (100%)	0	

N, number of cases; %. Percentage of cases; T, Tumor stage; N, lymph node deposits

**Table 4:** Silver Insitu Hybridization/Chromogen Insitu Hybridization (SISH/CISH) Scoring in HER2 Equivocal and Positive Immunoreactive Adenocarcinoma Cases

	SISH/ CISH	
	Amplified N (%)	Non amplified N (%)
HER2 score (N)		
Equivocal (3)	2 (66.7%)	1 (33.3%)
Positive (3)	3 (100%)	0 (0%)
* P*-value	0.273	
HER2 pattern (N)		
Cytoplasmic (2)	2 (100%)	0 (0%)
Cytoplasmic/ membranous (4)	3 (75%)	1 (25%)
* P*-value	0.439	

N, number of cases; %, Percentage of cases

**Table 5:** SKP2 Immunoreactivity among Studied Groups

Histopathological diagnosis (N)	Subtypes (N)	SKP2 expression		SKP2 pattern	
		Negative N (%)	Positive N (%)	Cytoplasmic N (%)	Cytoplasmic/nuclear N (%)
Adenocarcinoma (CRC) (40)		18 (45%)	22 (55%)a	6(33.3%)	12(66.7%)
	Conventional	17(53.1%)	15(46.9%)	6(40%)	9(60%)
	Low grade (20)	13 (65%)	7 (35%)	2 (28.6%)	5 (71.4%)
	High grade (12)	4 (33.3%)	8 (66.7%)	4(50%)	4 (50%)
	Mucinous (8)	5 (62.5%)	3 (37.5%)	0 (0%)	3 (100%)
Adenoma (15)		4(26.7%)	11(73.3%)a	1(25%)	3 (75%)
	Tubular type (7)	6(86%)	1(14%)	0	1 (100%)
	Tubulo-villous (8)	5(62.5%)	3(37.5%)	1(33.3%)	2 (66.7%)
	With Low grade dysplasia (6)	4(66.7%)	2(33.3%)	0	2 (100%)
	With high graded ysplasia (9)	7(77.8%)	2(22.2%)	1(50%)	1(50%)
Ulcerative colitis (UC) (15)	With low grade dysplasia (7)	15 (100%)	0	-	-
	No dysplasia (8)				
Colorectal hyperplastic polyps (8)		8 (100%)	0	-	-
Chronic non- specific colitis (19)	Moderate colitis (13)	19 (100%)	0	-	-
	Severe colitis (6)				
Bilharzial colitis (8)		8 (100%)	0	-	-
Minimal colitis / controls (22)		22 (100%)	0	-	-

N, number of cases; %. Percentage of cases; CRC, colorectal adenocarcinoma; UC, ulcerative colitis; ^a^ Crosstab, Pearson chi square (p<0.001) compared to different groups

**Table 6:** SKP2 Immunoreactivity in Resection CRC Cases

Stage		SKP2 expression			SKP2 pattern		
		Negative	Positive	*P *	Cytoplasmic	Cytoplasmic/nuclear	*P*
		N (%)	N (%)		N (%)	N (%)	
T	T3 (12)	5 (41.7%)	7 (58.3%)	0.732	1 (14.3%)	6 (85.7%)	0.201
	T4 (6)	2 (33.3%)	4 (66.7%)		2 (50%)	2 (50%)	
N	N0 (6)	1 (16.7%)	5 (83.3%)	0.393	2(40%)	3 (60%)	0.452
	N1 (6)	3 (50%)	3 (50%)		0	3 (100%)	
	N2 (6)	3 (50%)	3 (50%)		1 (33.3%)	2 (66.7%)	

N, number of cases; %, Percentage of cases; T, Tumor stage; N, lymph node deposits

## Discussion

Colorectal cancer (CRC) is characterized by compounding genetic mutations in both oncogenes and tumor-suppressor genes that drive its initiation and promotion under various pathophysiological conditions (Seigel et al., 2011). HER2 mutation is important for clinical treatment and prognosis evaluation in cancer patients and has been found to be a predictive marker to HER2-targeted therapy in breast and gastric cancer (Calhoun and Collins, 2015; Jiang et al., 2018). Several studies evaluating HER 2 in CRC resulted in a large debate because overexpression rates varied between zero and 84% (Osako et al., 1998). 

The current study was conducted on randomly collected archived paraffin blocks of 105 cases with different colonic lesions. In different studied groups, age ranged from 15-77 years with male predominance (51.5%) which was going with Bochis et al., (2017). The majority of CRC patients were males (57.5%) and mean age was 55 years. Two Egyptian studies by Mohamed and Tealeb, 2015 and Elwy et al., (2012) demonstrated similar results where 70.6% and 60% of their studied patients respectively were males and mean age was 51 and 49.3 years respectively. These results differ from what was reported by Cressey et al., (2006) who detected a higher incidence of CRC in females (63%). Furthermore, studies done by Terzi et al., (2008) and Office for National Statistics, 2011 reported CRC incidence in a higher age than ours (> 60 years). These differences may be due to random collection of cases and different etiologic and predisposing factors for CRC in Egypt compared to other countries.

Regarding HER2 immunoexpression; scores 2+ and 3+ were found only in15% of our CRC cases. This matches the results of Li et al., 2011. However, higher percentages were reported by Elwy et al., 2012 (23%) and Sayadnejad et al., 2017 (24%). A much higher percentage was reported by Kruszewski et al., 2010 (77%) and Shabbir et al., 2016 (78.9%). On the other hand, lower percentage was reported by Nathanson et al., (2003) (3.6% of American patients), Marx et al., (2010) (2.7% of German patients), Heppner et al., (2014) (1.6% of British patients). Meanwhile, AL-Kuraya et al., (2007) did not find any positivity in a study of 98 Saudi patients. The random selection and relative small sample size could explain the differences between studies.

We found a high statistical significant difference of HER2 expression in CRC group compared with other studied groups which were all negative. This goes with results of Heidari et al., (2017) who found a higher significant difference comparing CRCs to adenomas and normal tissues. Also, Pazurek et al., (2009) found lower expression of HER2 in colon adenoma compared with cancer group by using PCR method.

The food and drug administration (FDA) approved scoring system for breast cancer which is entirely based on membranous HER2 overexpression with strict guidelines to ignore cytoplasmic expression as it does not correlate to any clinical outcome (Walker et al., 2008). In contrast to breast cancer, there is evidence that in CRC; cytoplasmic HER2 could be associated with survival prognosis; as it may be involved in tumor pathogenesis like membranous HER2 in breast cancer (Blok et al., 2013). Intracellular HER2 targeting compounds might be attractive treatment option in one third of CRC patients where cytoplasmic HER2 is actively involved in carcinogenesis of CRC (Seo et al., 2014). 

Regarding subtypes of CRC, 5/6 of our positive and equivocal tumors were of conventional subtype but we found no statistically significant relationship between the subtype and HER2 pattern; which matches results of Kruszewski et al., (2010), Elwy et al., (2012) and Mohamed and Tealeb, (2015). In contrast to our results; Shabbir et al., 2016 observed more common membranous HER2 staining in mucinous CRC while more frequent cytoplasmic staining in non-mucinous types.

With respect to grade of differentiation in CRC cases, 4/6 of the positive and equivocal tumors were of low grade and exhibit mainly strong cytoplasmic/membranous expression, however no significant relationship was observed between CRC grades and HER2 pattern, which is consistent with several studies (Elwy et al., 2012; Pappas et al., 2013; Sayadnejad et al., 2017). However, several other studies reported the contrary. Half and his colleagues, 2004 found a significant relation between the cytoplasmic HER2 staining and tumor differentiation. Also, Shabbir et al., (2016) concluded a significant strong association between cytoplasmic HER2 expression and low grades of CRC, as well as between membranous HER2 expression and high grade CRC. 

In agreement with Song et al., (2014), Shabbir et al., (2016) and Sayadnejad et al., (2017) we did not find a significant link between HER2 expression and tumor stage or lymph node metastasis (TN stage). On the contrary, Elwy et al., (2012) and Heppner et al., (2014) reported a significant association with higher stages and positive nodal status. 

In our study, SISH and CISH for HER2 were applied on 6/40 CRCs. Theses 6 CRC specimens were expressing equivocal (3) and positive (3) staining by IHC. HER2 scoring demonstrated high concordance rates between dual-color SISH and CISH methods as the results of the two techniques were almost identical showing 5 out of these 6 cases i.e 5/40 CRCs (12.5%) proved true for *HER2* gene amplification. Our results were not far from studies of Valtorta et al., (2015) and Zhang et al., (2020) who reported that 5% and 5.63% of their CRC patients had HER2 amplification. Heppner et al., (2014) and Seo et al., (2014) stated that HER2 amplification ranged from 1.6% to 6.3%. However, a Korean study by Park et al., (2007) showed that HER2 expression rate was 47%. There are several possible reasons for this discrepancy such as ethnic diversity, technical variability in the IHC performance, sample size, heterogeneity of study population, racial differences, and varied experimental designs (Li et al., 2014; Seo et al., 2014). Another key subject is lack of agreement about whether only membranous, cytoplasmic or both should be considered for evaluation of HER2 overexpression (Pappas et al., 2013).

Researches about the SKP2 signaling suggest that SKP2 targeting may be a very attractive approach to treat human cancers. Chen et al., 2014 confirmed the hypothesis that SKP2 siRNA (small interference RNA) may be a useful therapeutic protocol for the treatment of colon carcinoma.

Our results showed a high statistically significant SKP2 expression in CRC group compared with other studied groups. In our study; 45% of CRC cases were positive to SKP2, this matches results of Li et al., (2004), Ni et al., (2009) and Tian et al., (2013) who found that 50%, 48% and 47.6% of their studied cases respectively expressing SKP2. 

No statistically significant relationship was detected between histologic subtypes of CRC and SKP2 immunostaining. This was the same finding of Uddin et al., (2008) on a large tissue microarray of 448 samples of mouse models.

With respect to the grade of CRC tumor differentiation, no significant relationship was observed in relation to SKP2 expression which was consistent with Bochis et al., (2017). On the contrary, Lu et al., (2009) and Shen et al., (2018) found SKP2 to be highly associated with histological grade of tumor as SKP2 was highly expressed in poorly differentiated CRCs.

In agreement with Shapira et al., (2005), we concluded that there was no statistically significant link between expression of SKP2 in CRC cases and tumor stage or nodal metastases (TN) stage. In contrast, TNM stage was significantly correlated with the expression of SKP2 in the study of Bochis et al., (2017).

Regarding adenoma group, SKP2 positivity was detected in 26.7%. However, Ni et al., (2009) found SKP2 positivity in only 5% of their adenoma cases. In agreement with our results, Li et al., (2004) and Ni et al., (2009) reported that SKP2expression was sequentially increased from normal mucosa through adenoma to primary carcinoma. 

Correlation of SKP2 expression in both types of adenoma (tubular and tubulovillous) and in different grades of associated dysplasia (low and high) showed no statistical significant difference. On the contrary, Li et al., (2004) found a significant increased SKP2 expression from mild through moderate to severe dysplasia in adenomas and these increases were confirmed by Western blot.

In a study done by Lui et al., (2012) on breast invasive carcinomas they found correlation between SKP2 and HER2 positivity. Our results showed a positive correlation between HER2 and SKP2 immunostaining in studied cases. To our knowledge, there is no other published studies examined the correlation between HER2 and SKP2 in benign or malignant colonic lesions.

In conclusion, the prognostic role of HER2 in CRC remains uncertain. Our findings may serve as a basis for future studies on patient selection for HER2 targeted therapy. Although a small percent of CRC patients exhibited* HER2 *gene amplification, these patients would be potential candidates for anti-HER2 therapy and IHC could be a primary screening test for patient selection. Additionally, more studies on the value of cytoplasmic HER2 expression in CRC must be done as if cytoplasmic HER2 has a pathophysiological role in CRC, intracellular HER2-targeting compounds might be a new treatment choice for these patients.

The potential carcinogenic role of SKP2 was suggested by the findings that SKP2 expression was undetectable in normal colon mucosa but significantly increases from adenoma to carcinoma, hoping these patients to get benefit from targeted therapy.

## Funding

This work was financed by TBRI internal project No.113T. Principal investigator: Prof. Dr. Mona Moussa.
